# Immune Profiles to Predict Response to Desensitization Therapy in Highly HLA-Sensitized Kidney Transplant Candidates

**DOI:** 10.1371/journal.pone.0153355

**Published:** 2016-04-14

**Authors:** Julie M. Yabu, Janet C. Siebert, Holden T. Maecker

**Affiliations:** 1 Department of Medicine, Stanford University School of Medicine, Palo Alto, CA, United States of America; 2 CytoAnalytics, Denver, CO, United States of America; 3 Institute for Immunity, Transplantation, and Infection, Stanford University School of Medicine, Palo Alto, CA, United States of America; University of Toledo, UNITED STATES

## Abstract

**Background:**

Kidney transplantation is the most effective treatment for end-stage kidney disease. Sensitization, the formation of human leukocyte antigen (HLA) antibodies, remains a major barrier to successful kidney transplantation. Despite the implementation of desensitization strategies, many candidates fail to respond. Current progress is hindered by the lack of biomarkers to predict response and to guide therapy. Our objective was to determine whether differences in immune and gene profiles may help identify which candidates will respond to desensitization therapy.

**Methods and Findings:**

Single-cell mass cytometry by time-of-flight (CyTOF) phenotyping, gene arrays, and phosphoepitope flow cytometry were performed in a study of 20 highly sensitized kidney transplant candidates undergoing desensitization therapy. Responders to desensitization therapy were defined as 5% or greater decrease in cumulative calculated panel reactive antibody (cPRA) levels, and non-responders had 0% decrease in cPRA. Using a decision tree analysis, we found that a combination of transitional B cell and regulatory T cell (Treg) frequencies at baseline before initiation of desensitization therapy could distinguish responders from non-responders. Using a support vector machine (SVM) and longitudinal data, *TRAF3IP3* transcripts and HLA-DR-CD38+CD4+ T cells could also distinguish responders from non-responders. Combining all assays in a multivariate analysis and elastic net regression model with 72 analytes, we identified seven that were highly interrelated and eleven that predicted response to desensitization therapy.

**Conclusions:**

Measuring baseline and longitudinal immune and gene profiles could provide a useful strategy to distinguish responders from non-responders to desensitization therapy. This study presents the integration of novel translational studies including CyTOF immunophenotyping in a multivariate analysis model that has potential applications to predict response to desensitization, select candidates, and personalize medicine to ultimately improve overall outcomes in highly sensitized kidney transplant candidates.

## Introduction

Kidney transplantation is the most effective treatment for end-stage kidney disease (ESRD) in terms of mortality, quality of life, and health care savings [1]. Sensitization, the formation of human leukocyte antigen (HLA) antibodies against a transplant, remains a major barrier to successful kidney transplantation. HLA antibodies are acquired through exposure to foreign HLA antigens, most commonly from previous transplants, pregnancies, and transfusions. After implementation of the new kidney allocation system one year ago, the number of transplants increased six-fold from 2–3% transplantation rate for the highly sensitized patients with cumulative calculated panel reactive antibody (cPRA) 99–100% (http://optn.transplant.hrsa.gov). However, the majority of highly sensitized patients fail to find a compatible donor and remain on dialysis. Desensitization strategies that utilize medications to nonspecifically target both HLA antibodies and underlying immune cells have allowed successful transplantation in only a relatively small proportion of highly sensitized candidates.

One limitation of desensitization therapy is that a significant number of candidates do not respond. Current progress is hindered by lack of in-depth immune monitoring strategies that can predict which candidates respond to therapy and can guide tailored desensitization strategies based on individual immune profiles. Furthermore, detailed mechanisms of how desensitization therapy modulates specific immune cell subpopulations and intracellular signaling pathways are poorly understood.

Our objective was to determine whether baseline differences in immune profiles could help identify those candidates that will respond to desensitization therapy. We report the application of single-cell mass cytometry time-of-flight (CyTOF) phenotyping, gene arrays, and phosphoepitope flow cytometry to study immune and gene expression profiles in a cohort of highly sensitized candidates undergoing desensitization therapy. The CyTOF platform, which uses antibodies labeled with heavy metal isotopes, allows the ability to simultaneously measure numerous parameters per cell at one time [2].

In this study, we used baseline and serial longitudinal samples of peripheral blood to prospectively follow immune profiles, gene expression, and key intracellular signaling pathways before and during desensitization therapy. We hypothesized that candidates who responded to desensitization therapy as measured by HLA antibodies would have a different immune and gene expression profile from those candidates who failed to respond.

## Materials and Methods

### Participants

20 participants with ESRD and cumulative cPRA 93–100% were treated with desensitization therapy. Participants were offered the protocol based on waiting time on the deceased donor kidney transplant list or availability of an incompatible living donor. All candidates voluntarily participated in the protocol. Written informed consent was obtained from all participants. The consent included HIPPAA authorization for access to medical records. The Institutional Review Board at Stanford University approved this protocol (numbers 15267 and 17997). Data from 138 age-matched healthy control samples (age range 25–66 years) were obtained from a previously performed flu vaccine study [3].

### Desensitization protocol

A modified version of the high-dose intravenous immunoglobulin (IVIG) and rituximab protocol previously reported by Vo and Jordan was used [4]. All candidates were treated with monthly IVIG at 2 gm/kg, maximum dose 140 gm, prior to single-dose rituximab 375 mg/m^2^ intravenously for at least six months. After 6–12 months, candidates were treated with bortezomib 1.3 mg/m^2^ intravenously every 72 hours for 4 doses and plasmapheresis for 1–2 cycles if there was lack of response to IVIG and rituximab per a modified version of a protocol previously reported by Woodle [5]. All candidates who underwent transplantation received the last dose of desensitization within one month of transplantation. After transplantation, all candidates were treated with anti-thymocyte globulin (ATG) induction therapy and mycophenolate mofetil (MMF), tacrolimus, and prednisone for maintenance immunosuppression.

### HLA antibody measurements

The response to therapy was assessed by decrease in cumulative cPRA, which represents a composite of HLA antibody levels and is an estimate of the probability of receiving a compatible transplant. HLA antibody screening was performed before and after IVIG therapy. Luminex HLA Class I and II single-antigen beads and Fusion analysis (LabScreen, One Lambda, Canoga Park, CA, USA) were used to determine HLA antibody specificities. HLA antibodies with ≥1000 normalized mean fluorescence intensity (MFI) were considered positive. cPRA was determined using HLA frequencies from United Network of Organ Sharing (UNOS) based on a recent cohort of deceased donors.

An acceptable cross match to proceed with transplantation after desensitization at our center was T-cell and B-cell flow cross match (FXM) with a median flow-channel shift (MCS) of ≤ 200 after adjusting for presence of autoantibodies (normal range MCS T-cell FXM ≤ 88 MCS and B-cell FXM ≤ 100 MCS). HLA antibodies were considered positive with normalized mean fluorescence intensity (MFI) of >1000 and “possible” with MFI 500–999. Our center threshold for listing unacceptable HLA antigens was 1000 MFI. However, in participants undergoing desensitization, we raised the threshold and listed HLA antigens as unacceptable with MFI strength 3000 or higher. All participants had post-transplant donor specific antibody (DSA) monitoring and protocol kidney biopsies at implantation and 3 and 12 months post-transplant.

### Outcome measures

The response to therapy was assessed by a predefined decrease of 5% or greater in cumulative cPRA. HLA antibody screening was performed before and after IVIG therapy or monthly. Non-response was defined as no decrease in HLA antibodies as measured by cPRA after all therapies were completed.

### Sample collection and processing

Serial samples of peripheral blood were collected to prospectively follow immune and gene expression profiles of candidates before and during desensitization therapy. Healthy control samples were previously obtained. Peripheral blood mononuclear cells (PBMC) for CyTOF phenotyping and phosphoepitope flow cytometry assays were isolated by Ficoll gradient centrifugation and cryopreservation as previously described [6]. For gene arrays, blood was collected directly into PAXgene Blood RNA Tubes (BD PreAnalytix) using a 21-gauge butterfly needle and catheter to ensure that tubes could be held low and vertical to maintain the vacuum on top of the stabilization solution. Tubes were inverted 10 times after drawing. Samples were incubated in collection tubes at room temperature for at least 2 hours, then stored at -80°C within 4 hours.

### CyTOF immunophenotyping

This assay was performed in the Human Immune Monitoring Center (HIMC) at Stanford University as previously described [2]. PBMC were thawed in warm media, washed twice, resuspended in CyFACS buffer (PBS supplemented with 2% BSA, 2 mM EDTA, and 0.1% sodium azide), and viable cells were counted by Vicell. Cells were added to a V-bottom microtiter plate at 1.5 million viable cells/well, washed once by pelleting, and resuspended in fresh CyFACS buffer. The cells were stained for 60 minutes on ice with 50 μL of the following antibody-polymer conjugate cocktail (Table in S1 Table). All antibodies were from purified unconjugated, carrier-protein-free stocks from BD Biosciences, Biolegend, or R&D Systems. The polymer and metal isotopes were from Fluidigm. The cells were washed twice by pelleting and resuspended with 250 μL FACS buffer. The cells were resuspended in 100 μL PBS buffer containing 2 μg/mL Live-Dead (DOTA-maleimide (Macrocyclics) containing natural-abundance indium). The cells were washed twice by pelleting and resuspended with 250 μL PBS. The cells were resuspended in 100 μL 2% PFA in PBS and placed at 4°C overnight. The next day the cells were pelleted and washed by resuspension in fresh PBS. The cells were resuspended in 100 μL eBiosciences permeabilization buffer (1x in PBS) and placed on ice for 45 minutes before washing twice with 250 μL PBS. The cells were resuspended in 100 μL iridium-containing DNA intercalator (1:2000 dilution in PBS; Fluidigm) and incubated at room temperature for 20 minutes. The cells were washed twice in 250 μL MilliQ water. The cells were diluted in a total volume of 700 μL in MilliQ water before injection into the CyTOF system (Fluidigm). Data analysis was performed using FlowJo v9.3 (CyTOF settings) by gating on intact cells based on the iridium isotopes from the intercalator, then on singlets by Ir191 versus cell length, then on live cells (Indium-Live-Dead minus population), followed by cell subset-specific gating.

### Gene expression profiling

Work was performed in the HIMC at Stanford University. Total RNA was isolated according to the manufacturer’s instructions by using a PAXgene RNA blood kit (Qiagen). The entire procedure was carried out at room temperature with the QIAcube automated robot (Qiagen). Total RNA yield was assessed using the Thermo Scientific NanoDrop 1000 micro-volume spectrophotometer (absorbance at 260 nm and the ratio of 260/280 and 260/230). RNA integrity was assessed using the Agilent’s Bioanalyzer NANO Lab-on-Chip instrument (Agilent).

Cy3-labeled, amplified antisense complementary RNA (cRNA) targets were prepared from 20 to 500 ng of the total RNA using the QuickAmp Labeling kit or the Low Input Quick Amp Labeling Kit (Agilent). 850 μg of labeled cRNA was hybridized overnight to Agilent Whole Human Genome 4 x 44 K slides, which contain 44,000 probes, including 19,596 Entrez Gene RNAs. The arrays were then washed, blocked, stained, and scanned on the Agilent microarray scanner following the manufacturer’s protocols.

### Phosphoepitope flow cytometry

This assay was performed in the HIMC at Stanford University. PBMC were thawed in warm media, washed twice, and resuspended at 0.5x10^6^ viable cells/ml. 200 μL of cells were plated per well in 96-well deep well plates. After resting for 1 hour at 37°C, cells were stimulated by adding 50 μl of cytokine (IFN-alpha, IFN-gamma, IL-6, IL-7, IL-10, IL-2, or IL-21) and incubated at 37°C for 15 minutes. PBMC were then fixed with paraformaldehyde, permeabilized with methanol, and kept at -80°C overnight. Each well was barcoded using a combination of Pacific Orange and Alexa-750 dyes (Invitrogen, Carlsbad, CA) and pooled in tubes. The cells were washed with FACS buffer (PBS supplemented with 2% FBS and 0.1% sodium azide) and stained with the following antibodies (all from BD Biosciences, San Jose, CA): CD3 Pacific Blue, CD4 PerCP-Cy5.5, CD20 PerCp-Cy5.5, CD33 PE-Cy7, CD45RA Qdot 605, pSTAT-1 AlexaFluor488, pSTAT-3 AlexaFluor647, and pSTAT-5 PE. The samples were then washed and resuspended in FACS buffer. 100,000 cells per stimulation condition were collected using DIVA 6.0 software on an LSRII flow cytometer (BD Biosciences). Data analysis was performed using FlowJo v9.3 by gating on live cells based on forward versus side scatter profiles, then on singlets using forward scatter area versus height, followed by cell subset-specific gating. 90^th^ percentile fluorescence values for each pSTAT readout/cytokine/cell subset combination were used to derive fold-change relative to the unstimulated pSTAT readout for that cell subset.

### Informatics

#### Baseline immunophenotypes

Differences in 48 immunophenotypes between responders and non-responders prior to treatment were compared using *t*-tests. Support vector machines (SVMs) were built using the e1071 package in R (type = C-classification, kernel = linear). A decision tree was built using the J48 decision tree algorithm in Weka [7].

#### Gene expression pre-processing

Data was processed with the R packages affyPLM and limma [8]. Expression levels were background corrected with RMA, normalized with quantile normalization, and summarized using the average log2 value across all probes in the probe set. Hereafter, all probe sets are called probes. Next, we fit a linear model for each probe using duplicateCorrelation and lmFit, estimating and accounting for the correlation between repeated measurements for each person. Then, we removed promiscuous probes that mapped to more than one gene and limited the analysis to the probe with the highest average expression for each gene (n = 18,566). Finally, we used the empirical Bayes method to smooth the standard errors (eBayes) and estimate the significance of the difference in expression between responders and non-responders using a moderated *t*-test [9]. The top 100 genes were retained for subsequent analysis.

#### Longitudinal analysis

Linear mixed models (analyte ~ response + sequence + response x sequence, with a random effect for each patient) were created for the top 100 genes, 48 CyTOF immunophenotypes, and 189 phosphoepitope flow cytometry analytes (which are a combination of cell population and cytokine stimulation, e.g. CD8+: pSTAT3.IFNg), using the nlme package in R [10]. Models were then ranked by the p-value on the response coefficient within assay to identify the two best models for each assay. P-values were adjusted with the false discovery rate (FDR) method.

#### Cross-assay analysis

To explore cross-assay relationships, we looked for significant associations between pairs of analytes, while accounting for response status. Data from all pre-transplant samples (n = 56) for which we had any combination of CyTOF phenotyping, gene expression, or phosphoepitope flow cytometry data was included. Analytes were the same as described in the longitudinal analysis. We built a total of 56,616 linear models of the form analyte1 ~ analyte2 + response. These regression models did not account for the repeated measurements from each patient. Forty-two percent of these were comprised of analyte pairs from different assays. We then filtered this data to those in which both the association between the analytes and the difference between responders and non-responders were statistically significant (p<0.001). To remove outliers, phosphoepitope flow cytometry data were limited to those analytes for which the mean fold change was ≥2 and the maximum fold change was ≤7. This yielded 93 cross-assay analyte pairs.

To search for classifiers, we attempted to classify the responders and the non-responders based on each analyte pair using a SVM algorithm. We built a total of 93 SVMs, as described above. The 93 analyte pairs spanned 72 unique analytes. We illustrated the resulting network and computed network statistics using the arc diagram package in R. Starting with these 72 analytes identified above, we built an elastic net logistic regression model to predict response status (non-responder = 0, responder = 1), using the glmnet package in R [11]. Data was scaled prior to the computation of the elastic net.

## Results

We analyzed immune and gene expression profiles from peripheral blood drawn from 20 candidates who underwent desensitization therapy, 10 responders and 10 non-responders. Demographic, clinical characteristics, histocompatibility data, immunosuppression and clinical outcomes of the participants are summarized in Table 1.

**Table 1:** Participant demographic, histocompatibility data, immunosuppression and clinical outcomes.

	Responders (n = 10)	Non-responders (n = 10)
Age (yr)	40 ± 13	44 ± 7
Male sex (%)	6 (60)	3 (30)
Transfusions (%)	10 (100)	10 (100)
Pregnancies (%)	4 (100)	4 (57)
Previous transplant (%)	6 (60)	6 (60)
Cause of ESRD		
Diabetes	1	1
Glomerulonephritis	0	1
SLE	0	2
PKD	3	1
Other	1	5
Cumulative cPRA (%)	97.1 ± 2.7	100 ± 0
Class I cPRA (%)	80.3 ± 23.8	98 ± 2.6
Class II cPRA (%)	70 ± 22.8	83.3 ± 27.6
Cumulative cPRA (%) after desensitization	87.7 ± 3.9	100 ± 0
FXM positive (%)	10 (100)	1 (33)
DSA positive (%)	10 (100)	1 (33)
IVIG (%)	1 (10)	0
IVIG/Rituximab (%)	4 (40)	5 (50)
IVIG/Rituximab/Bortezomib and Plasmapheresis (%)	5 (50)	5 (50)
Transplanted (%)	10 (100)	3 (30)
Donor type: DD:LUR:LRD	8:1:1	2:1:0
Borderline acute rejection	3 (30)	1 (33)
Cell mediated rejection	0	0
Antibody mediated rejection	0	0
Graft loss (%)	0	0
Death-censored graft survival (1 yr)	100%	100%

SLE = systemic lupus erythematosus; PKD = polycystic kidney disease; FXM = flow cross match; DSA = donor specific antibody; MFI = mean fluorescent intensity; DD = deceased donor; LUR = living unrelated; LRD = living related donor

For ten responders, FXM were positive (T-cell 88–200 MCS and B-cell 100–200 MCS) with DSAs present (500–1500 MFI). Two non-responders received 0 HLA-mismatched deceased donor kidney transplants with negative FXM. One non-responder received a living donor through paired donor exchange with a positive FXM. Seven non-responders failed to receive transplants. Strength of DSA was between 500–1500 MFI. Although the incidence of borderline rejection and graft outcomes were similar in responders and non-responders, only 30% of the non-responders were transplanted as a result of compatible, HLA-matched donors (two from 0 HLA-mismatched kidney transplants and one from paired donor exchange).

### Baseline immunophenotyping

Our initial analyses focused on immunophenotyping at baseline prior to the start of desensitization therapy. We performed CyTOF phenotyping using a panel of 33 antibodies (Table in S1 Table) to cover a broad spectrum of immune cell subsets as well as markers of activation and differentiation/exhaustion. Analysis for a total of 48 cell subsets (Table in S2 Table) yielded only two immune phenotypes with significant differences between responders and non-responders (data in S1 Fig). The cell subsets, CD94+CD8+ T cells and HLA-DR-CD38+CD8+ T cells, were significant at p<0.02 and p<0.05, respectively, without correction for multiple comparisons. These findings were not statistically significant after multiple comparison correction. Furthermore, by inspection, neither subset could be used to classify responders and non-responders. Thus, we applied machine learning to determine whether combinations of analytes could better segregate responders and non-responders.

**Fig 1:**
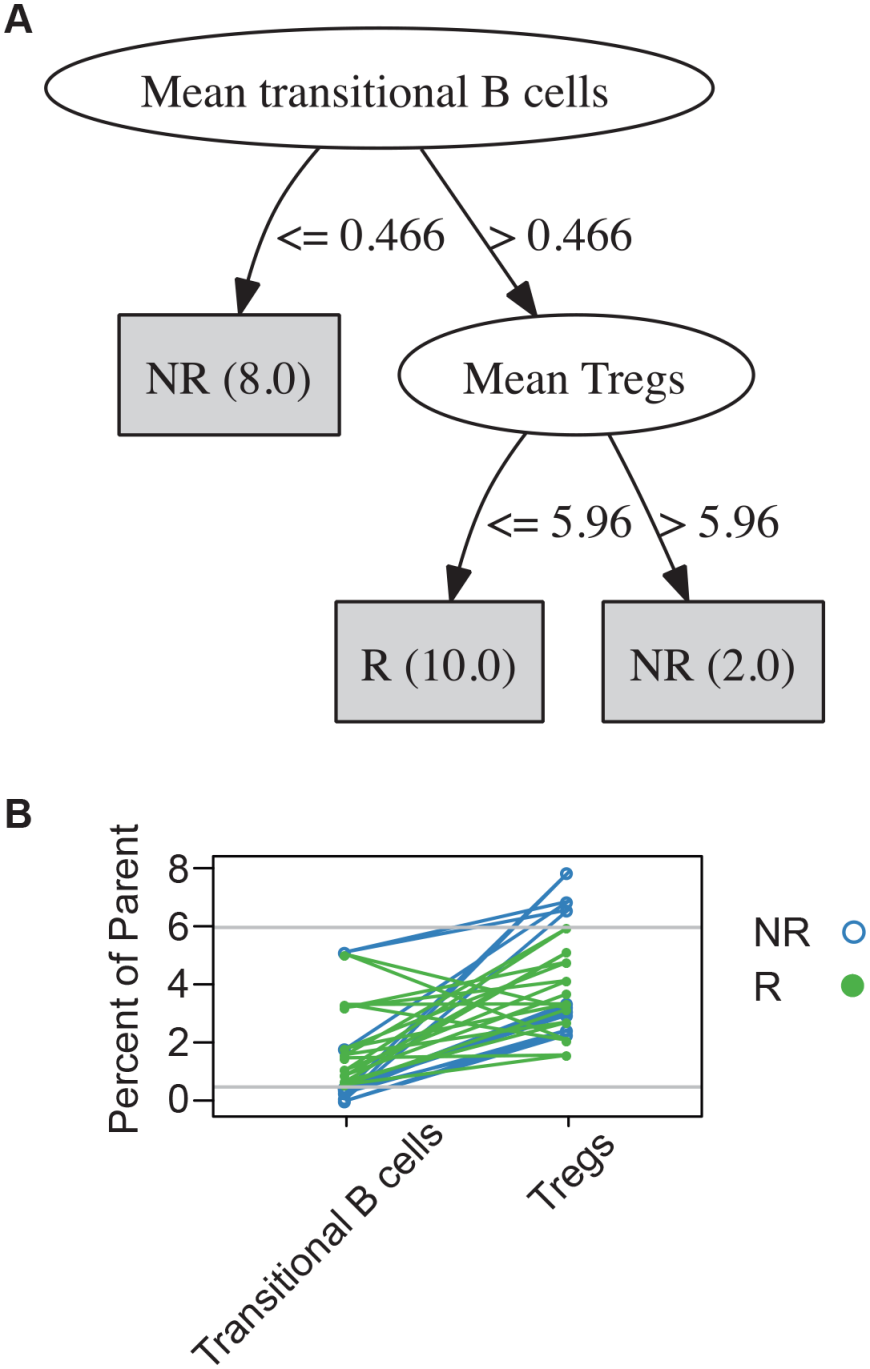
Levels of transitional B cells and Tregs classify responders and non-responders. (A) Decision tree algorithm illustrating the classification of responders (R; n = 10) and non-responders (NR; n = 10) based on two phenotypes. Numbers in parentheses are the numbers of patients who reach each leaf node. The leaf nodes are the shaded rectangular boxes. (B) Transitional B cells and Tregs by candidate and response group. Each line indicates one candidate. Horizontal reference lines indicate decision tree threshold values of 0.466 (transitional B cells) and 5.96 (Tregs).

We first used a support vector machine (SVM) algorithm to identify pairs of analytes that could classify responders and non-responders. Three of twenty classification errors were identified in all of the best resulting pairs from the SVM algorithm. A representative example (of six) is shown in data in S2 Fig. We next used a different machine-learning algorithm that generated a decision tree based on sequential cutoffs to classify samples according to outcome. Using this algorithm, two cell subsets, transitional B cells (CD14-CD33-/CD3-/CD19+CD20+/CD24+CD38+) and regulatory T cells (Tregs; CD14-CD33-/CD3+/CD4+/CD25hiCD127low), could classify with 100% accuracy (Fig 1).

**Fig 2:**
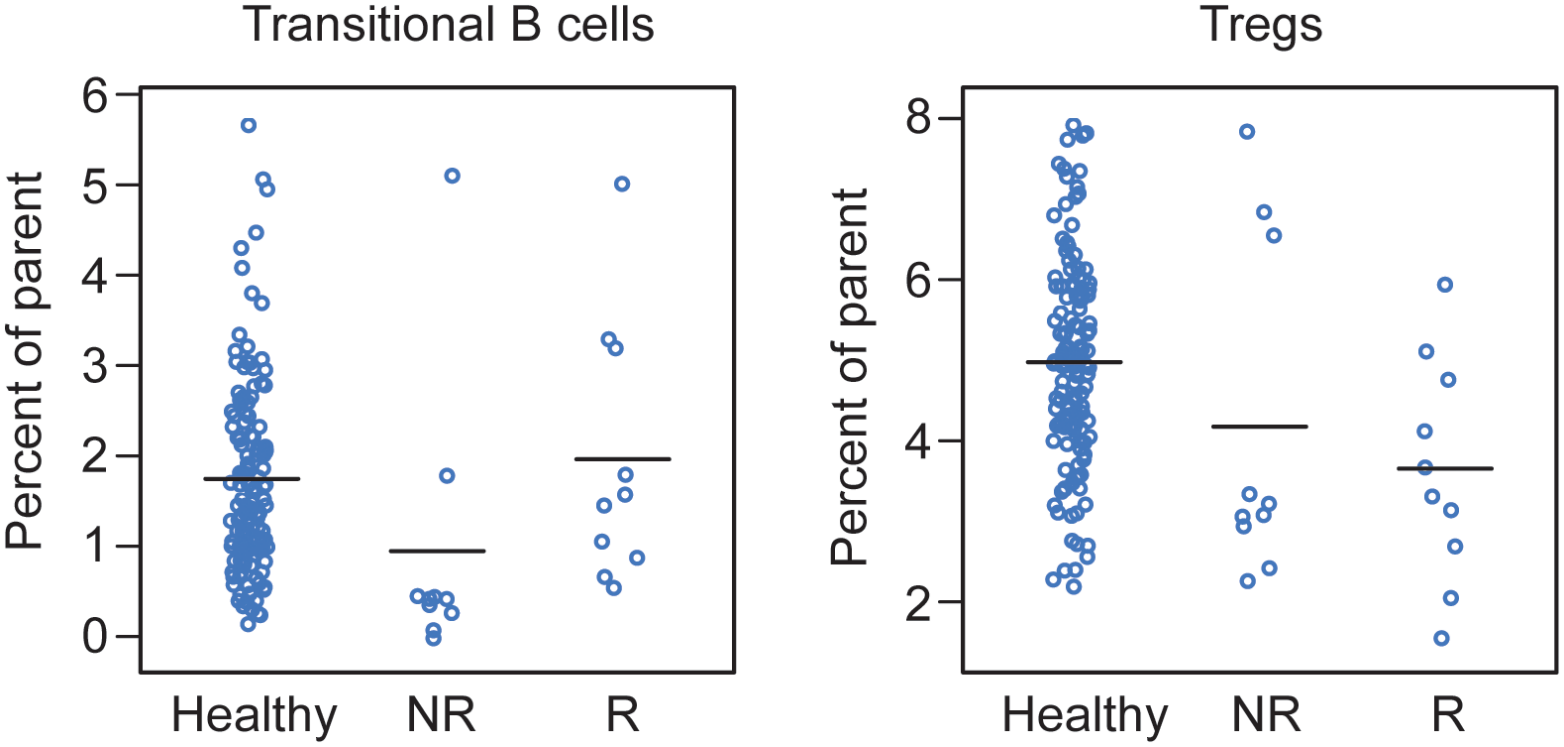
Transitional B cells and Tregs scatter plots compared to healthy controls. The values of transitional B cells and Tregs for transplant candidates appear largely distributed at the extremes of the healthy control population. This comparison illustrates that the thresholds selected by the decision tree tend toward the extremes in a much larger sample. Healthy = healthy controls; NR = non-responder; R = responder.

Given the small sample size, one possibility is that the decision tree outcome could be an artifact of this particular data set. In order to overcome the sample size and gain confidence in the biological significance of these two analytes, we compared the distribution of transitional B cells and Tregs in the transplant candidates to age-matched healthy controls. Data from 138 age-matched controls analyzed with the same CyTOF assay were compared in Fig 2. These results showed that the values for both transitional B cells and Tregs for all transplant candidates (responders and non-responders) tended to be skewed toward the extremes of the healthy control population. In fact, the threshold values chosen by the decision tree are at the extremes of the healthy control population.

### Longitudinal analyses

We next asked whether longitudinal analyses could improve the predictive power of immunophenotypes to classify responders and non-responders. In addition, we wanted to study the stability of the baseline findings over the course of therapy. We therefore performed CyTOF phenotyping, gene expression arrays, and phosphoepitope flow cytometry (abbreviated “Pheno,” “G,” and “Phospho,” respectively) on up to eight serial samples from baseline, pre-treatment, to 12 months during desensitization therapy on 10 candidates (five responders and five non-responders). We report here the two analytes with the lowest p-values for each assay.

**Fig 3:**
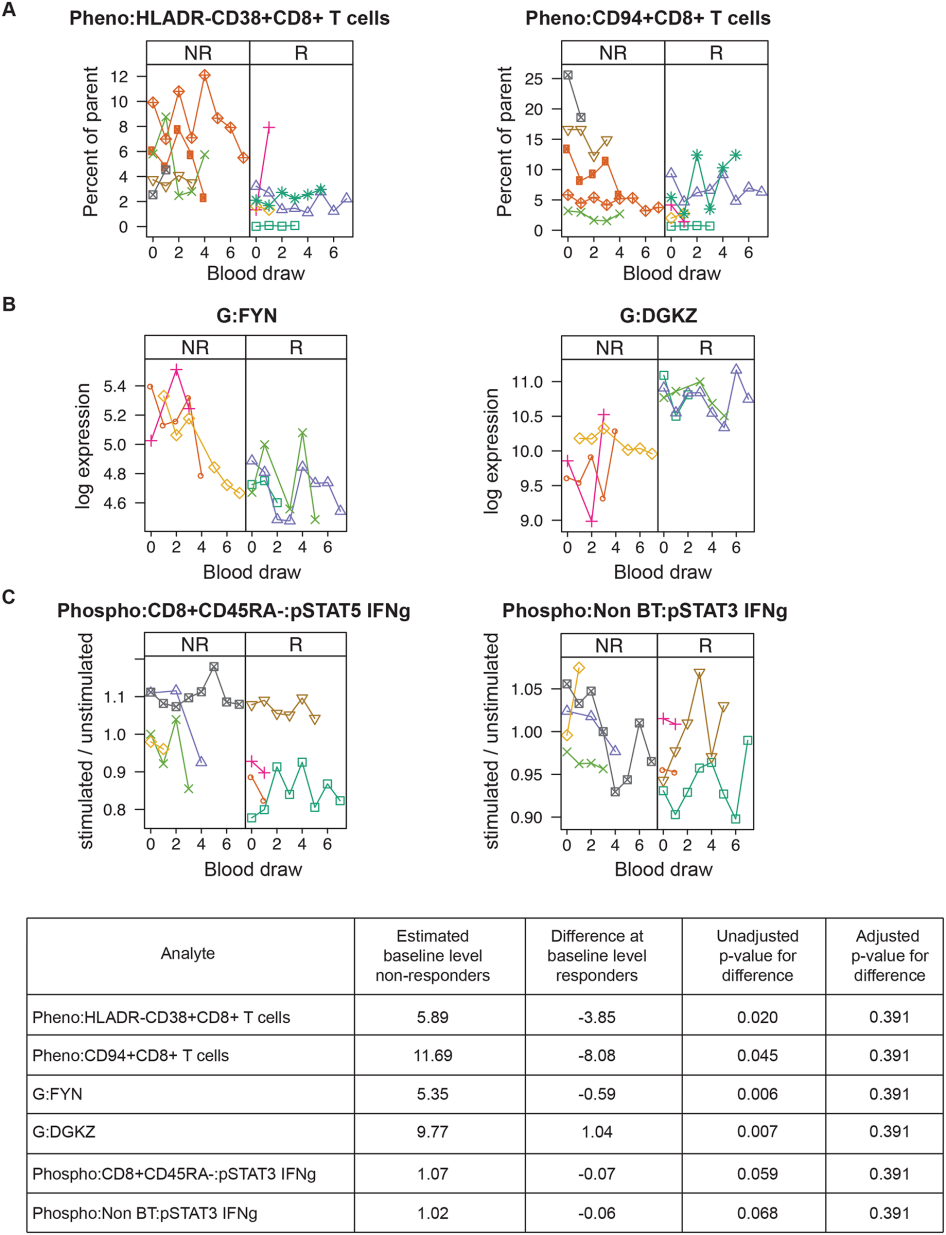
Top two longitudinal models by assay. (A) CyTOF phenotyping, (B) gene expression, (C) phosphoepitope flow cytometry. (Pheno = CyTOF phenotyping; G = gene expression; Phospho = phosphoepitope flow cytometry). Top models have the smallest p-values for the difference at baseline between responders and non-responders. Each symbol type and the connecting lines represent the values for one candidate across multiple blood draws. Blood draws are ordered by sequence number. Non-responders (NR) are in the left-hand panel of each figure, and responders (R) are in the right-hand panel. The table provides key metrics for the analyses. “Difference at baseline for responders” indicates the amount that the estimated average baseline level for the responders differs from that of the non-responders. “Unadjusted p-value for difference” and “Adjusted p-value for difference” are the unadjusted and adjusted p-values for this estimate, respectively.

For CyTOF phenotyping, HLADR-CD38+CD8+ T cells and CD94+CD8+ T cells, on average, were lower in responders versus non-responders (Fig 3A). For gene expression assays, the top two genes, *FYN* and *DGKZ*, showed relative stability over time. Levels of *DGKZ* were consistently higher in responders compared to non-responders (Fig 3B). Conversely, for the phosphoepitope flow cytometry assays, there were no significant differences between responders and non-responders with all the samples centered around 1 on the y-axis, indicating little or no reaction to cytokine stimulation (Fig 3C). While none of the p-values were significant after adjustment for multiple comparisons, the CyTOF phenotyping and gene expression data may suggest avenues of exploration in future studies.

### Cross-Assay Associations

**Fig 4:**
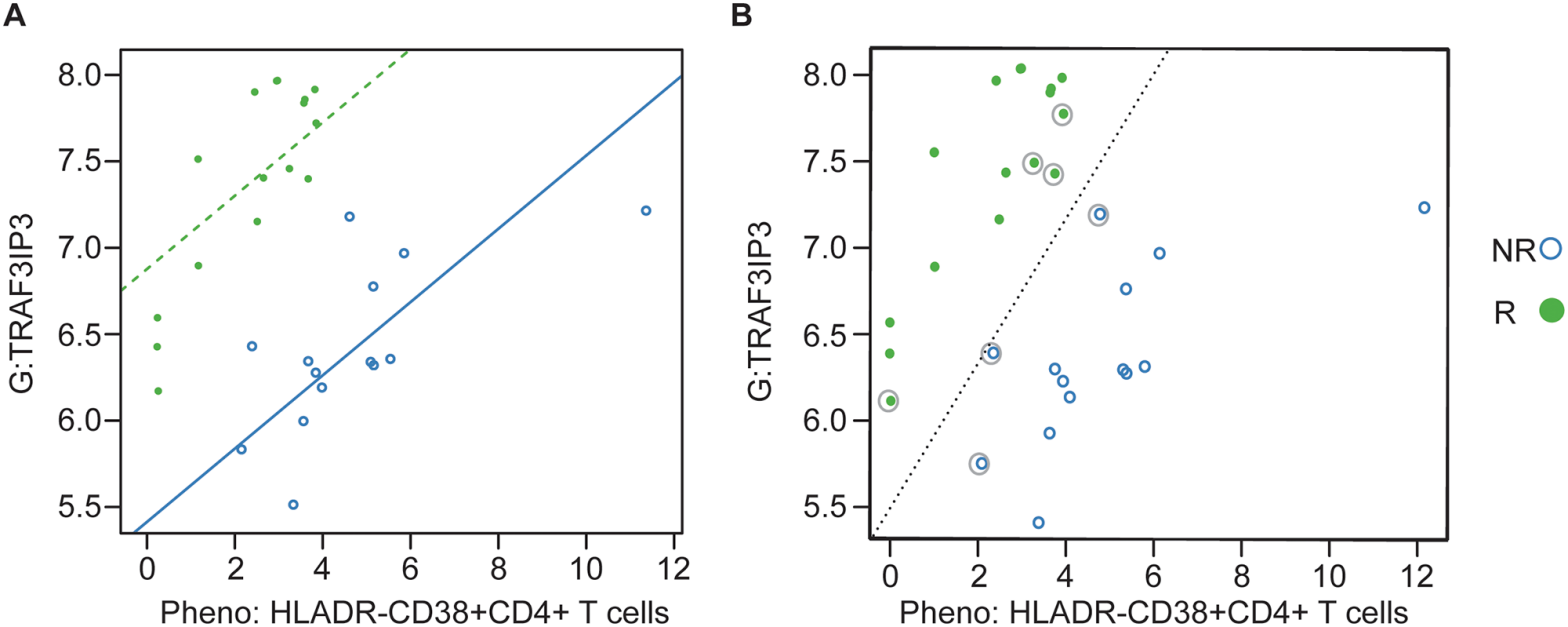
Best performing cross-assay pair using longitudinal samples. (A) Regression model with the solid blue line indicating the estimated relationship between the two analytes for non-responders (NR) and the dotted green line the relationship for responders (R). (B) Support vector machine (SVM) with the dotted line indicating the best linear relationship separating the two groups. Observations that are circled are the “support vectors,” the observations that drive the placement of the line of separation. All observations are correctly classified by the SVM. G = gene expression; Pheno = CyTOF phenotyping.

To better elucidate the possible interactions between analytes and across assays, we focused on cross-assay models (each analyte from a different assay) in which the p-value for both the correlation between the analytes, and the p-value for the difference between responders and non-responders, were both less than 0.001 (n = 93). Using an SVM, we found one combination of a cell subset frequency, HLA-DR-CD38+CD4+ T cells, and a gene expression level, *TRAF3IP3*, yielded perfect classification of responders and non-responders (Fig 4). Four additional pairs yielded only a single misclassified data point (data in S3 Fig).

**Fig 5:**
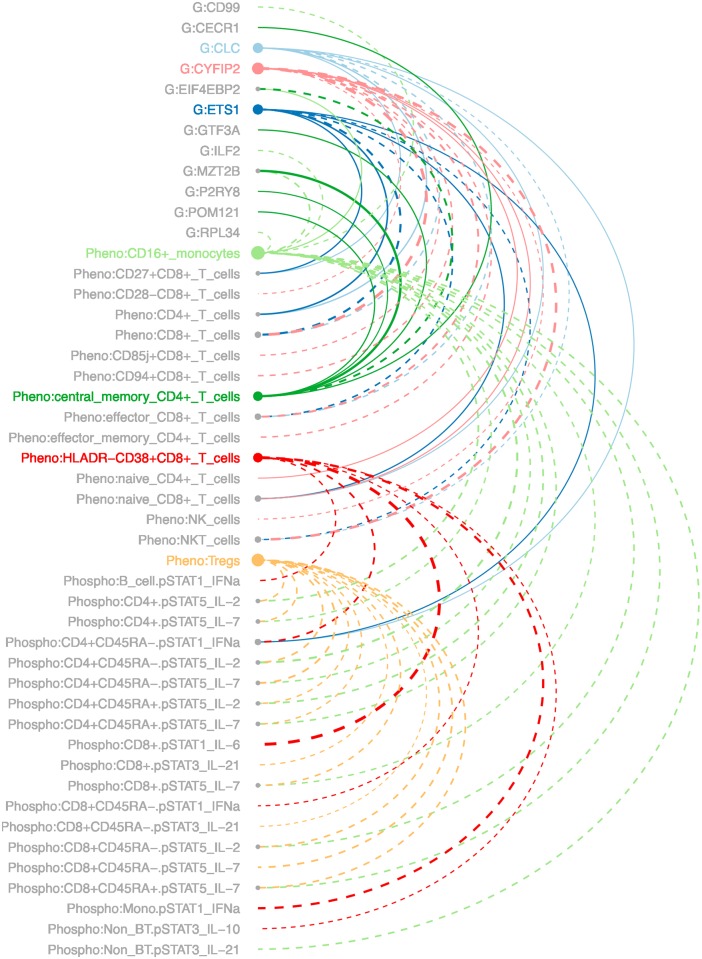
Network of highly connected analytes. Analytes are grouped by assay and listed alphabetically within assay. Node size indicates the number of connections for a particular analyte. Solid lines indicate negative correlation between the analytes, and dotted lines indicate positive correlation. Colors other than gray indicate the highly connected analytes. Analytes with names that are in gray are associated with one of the highly connected analytes. G = gene expression; Pheno = CyTOF phenotyping; Phospho = phosphoepitope flow cytometry.

The 93 pairs of analytes described above spanned 72 unique analytes. Of these, seven were highly connected, having at least six associations as described above. The resulting network included these highly connected analytes, and the analytes to which they are connected, for a total of 47 analytes. An arc diagram of the network is shown in Fig 5. As a whole, this suggests a high degree of relatedness between a few key genes and a subset of immunological readouts. These relationships might provide insights into the biological mechanisms of responsiveness to desensitization therapy.

**Fig 6:**
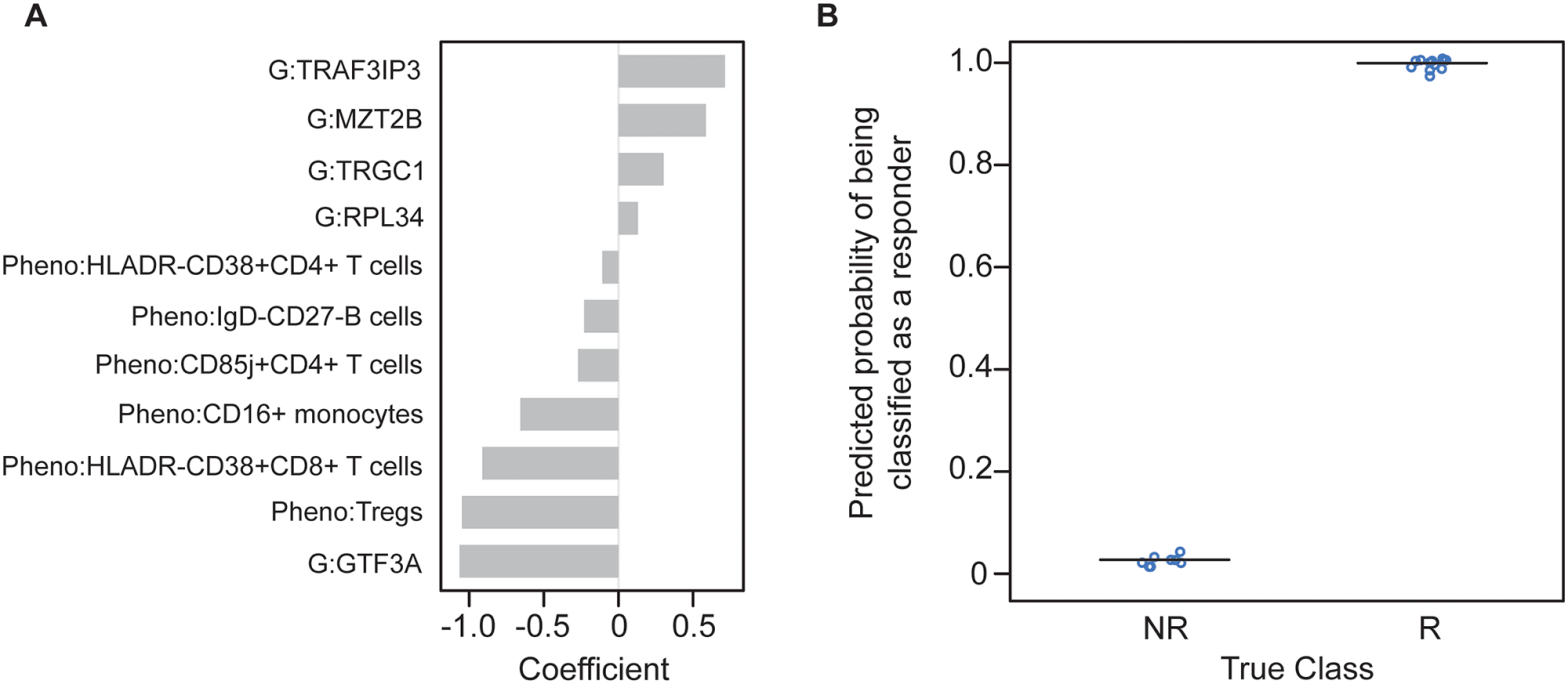
Elastic net logistic regression model. The model was built using the pairs of highly connected analytes shown in Fig 5. (A) Values of the coefficients for the 11 analytes included in the model. All data are normalized during the modeling. Thus, the values represent the number of standard deviations from the mean for each particular analyte. (B) Predicted probability of each observation being classified as a responder (R) or as a non-responder (NR). In this limited data set, the predicted probabilities are clearly separated and accurately predict response status. Since the non-responders for whom we had longitudinal multi-assay data were all in the high Tregs group, the large negative coefficient on Tregs is not unexpected. G = gene expression; Pheno = CyTOF phenotyping.

Starting with the 72 analytes identified by this systems immunology screen, we built an elastic net logistic regression model to predict response status. The model and its predictions are shown in Fig 6. The model, using 11 of the 72 analytes, provides impressive segregation between responders and non-responders.

## Discussion

Here we report for the first time the application of multi-assay immune monitoring, including mass cytometry, in highly sensitized kidney transplant candidates undergoing desensitization therapy to lower HLA antibodies and enable transplantation. This study was designed to determine if comprehensive immune profiling and the development of a multivariate analysis model might help predict which candidates respond to desensitization therapy.

First, we found significant baseline, pre-treatment differences between responders and non-responders. In our multivariate, decision tree model, we found that candidates with low numbers of transitional B cells and high numbers of Tregs were the most likely to not respond to therapy. Transitional B cells are seen in operationally tolerant patients and are thought to have regulatory and tolerogenic properties [12–14]. In addition, there is belief that tolerogenic B cells could balance allosensitization [15]. High levels of Tregs may represent an underlying inflammatory state secondary to infection or other ongoing immune events, leading to poor response to desensitization therapy. Notably, the thresholds set for the decision tree algorithm were at the extremes of the values of the normal healthy population. Therefore, a combination of baseline levels of immune phenotypes may predict response to therapy.

Second, we analyzed the trend of immune phenotypes, gene arrays, and phosphoepitope flow cytometry longitudinally after starting desensitization therapy. The top two analytes, HLADR-CD38+CD8+ T cells and CD94+CD8+ T cells, in which the responders were lower than non-responders were both markers of effector T cells. Interestingly, CD94/NKG2 is a lectin expressed predominantly on natural killer (NK) cells and a subset of CD8+ T cells [16]. NK-cell antibody-dependent cell mediated toxicity (ADCC) may contribute to microvascular damage associated with antibody-mediated rejection and may activate B cells via IFN-gamma or direct cell to cell contact [17]. In transplantation, NK cells are thought to promote either rejection or tolerance, and, therefore, it is unclear whether the presence of NK cells is beneficial or deleterious [18]. For our model, we also identified the top two genes and phosphoepitope flow signaling pathways that could separate responders from non-responders over many months of therapy. Although the p-values were not significant after adjustment for multiple comparisons, the longitudinal findings confirmed the stability of our baseline measurements, which did not change dramatically over the course of several time points during desensitization therapy. Therefore, this analysis provided evidence that baseline measurements can be utilized for our predictive model.

Finally, we combined all the assays and 72 analytes in multivariate analyses to determine the interrelatedness of different immune and biological markers and to identify top markers that can classify responders from non-responders. We found that the combination of an activated T cell phenotype, HLA-DR-CD38+CD4+ T cells, and the gene, *TRAF3IP3*, could perfectly separate responders from non-responders. *TRAF3IP3*, tumor necrosis factor-associated factor3 (TRAF3) interacting protein 3, mediates cell growth by up-regulating the c-Jun N-terminal kinase (JNK) pathway [19]. Moreover, *TRAF3PIP3* is specifically expressed in immune organs and tissues and may play a role in T and/or B cell development [19, 20]. Furthermore, we were able to examine the relationships between key genes and immune phenotypes and to develop a model of eleven analytes to predict response status. This model will need to be validated in a larger cohort.

Desensitization therapy is utilized to decrease HLA antibodies and the underlying immune cells responsible for antibody production. The exact mechanism of how these medications work or how to predict who responds to therapy is unknown. IVIG may exert its effects by binding to Fc receptors on immune cells, inhibiting IgG production, or inducing B cell apoptosis [21]. Rituximab, a chimeric CD20 monoclonal antibody, is postulated to decrease the production of HLA antibodies through targeting memory and naïve B cells without having any known effect on plasma B cells [22, 23]. However, memory B cells may be resistant to rituximab. Plasmaphereis removes plasma proteins including HLA antibodies without any known effect on B cells [24]. Bortezomib, a proteasome inhibitor, results in apoptosis of plasma cells although the effect on memory B cells and plasmablasts are unknown [5, 25]. Therefore, quantifying and measuring immune profiles to understand how desensitization therapies work or to predict who responds to treatment can provide valuable information for patient management.

This study is one of the first comprehensive analyses of immune profiling using CyTOF immunophenotyping in patients undergoing desensitization therapy. One of the strengths of this study is the use of three assays, each of which interrogates a different compartment of immune response. Another strength is its single center design that allows uniform desensitization and immunosuppression protocols and sample processing, prospective and consistent HLA antibody monitoring, and long-term and careful patient follow-up. As this is a single center study, one limitation is the small sample size, although the main objective of this pilot study was to develop a predictive model. Larger studies will need to be performed for validation.

In conclusion, this study of immune profiles in highly sensitized kidney transplant candidates shows that patients that respond to desensitization therapy with a reduction in HLA antibodies may have different baseline immune and gene expression profiles than those that are not responsive to therapy. This study demonstrates the translation of novel technology, comprehensive immune phenotyping, and analytic models to a challenging clinical setting of highly sensitized kidney transplant candidates. Ultimately, profiling of select immune phenotypes and key genes may permit the development of predictive biomarkers that can improve kidney transplant rates and outcomes in highly sensitized kidney transplant candidates.

## Supporting Information

S1 Fig Scatter plots of two baseline immune phenotypes. Non-responders (NR) and responders (R) show significantly different levels of CD94+CD8+ T cells and HLA-DR-CD38+CD8+ T cells at baseline (top row, n = 20). These frequency differences were concordant with differences in absolute cell counts (bottom row, n = 19), although there was no significant difference in the counts.
(TIF)

S2 Fig Pairwise analysis of baseline immune phenotype frequencies using support vector machines. The dotted line represents the support vector, or the line that best separates responders and non-responders. The most discriminating analyte pairs, including this representative (HLA-DR-CD38+CD8+ T cells and CD27+CD8+ T cells) misclassify three samples (green points above the dotted line) in this example. Pheno = CyTOF phenotyping.
(TIF)

S3 Fig Analysis of cross-assay pairs using support vector machines (SVM). Four pairs of analytes yielding an SVM with one classification error. The dotted line represents the support vector, or the line that best separates responders and non-responders. Observations that are circled are the “support vectors,” the observations that drive the placement of the line of separation. G = gene expression; Pheno = CyTOF phenotyping.
(TIF)

S1 Table CyTOF Antibody Immunophenotyping Panel. (DOCX)

S2 Table CyTOF Cell Subsets and Gating Pathway. (DOCX)

## Abbreviations

cPRA: calculated panel reactive antibody
CyTOF: single-cell mass cytometry time-of-flight
*DGKZ*: diacylglycerol kinase, zeta
ESRD: end-stage renal disease
FACS: fluorescence-activated cell sorting
FXM: flow cross match
*FYN*: tyrosine-protein kinase Fyn of the Src family
G: gene expression
HLA: human leukocyte antigen
*JNK*: c-Jun N-terminal kinase
Pheno: CyTOF phenotyping
Phospho: phosphoepitope flow cytometry
RMA: robust multi-array average
SVM: support vector machine
*TRAF3IP3*: tumor necrosis factor-associated factor3 (TRAF3) interacting protein 3
Treg: regulatory T cell
