# Supplementary figures and images for: Immune Profiles to Predict Response to Desensitization Therapy in Highly HLA-Sensitized Kidney Transplant Candidates
Source: PLoS One. 2016 Apr 14;11(4):e0153355. doi: 10.1371/journal.pone.0153355 (PMC4831845; doi:10.1371/journal.pone.0153355)

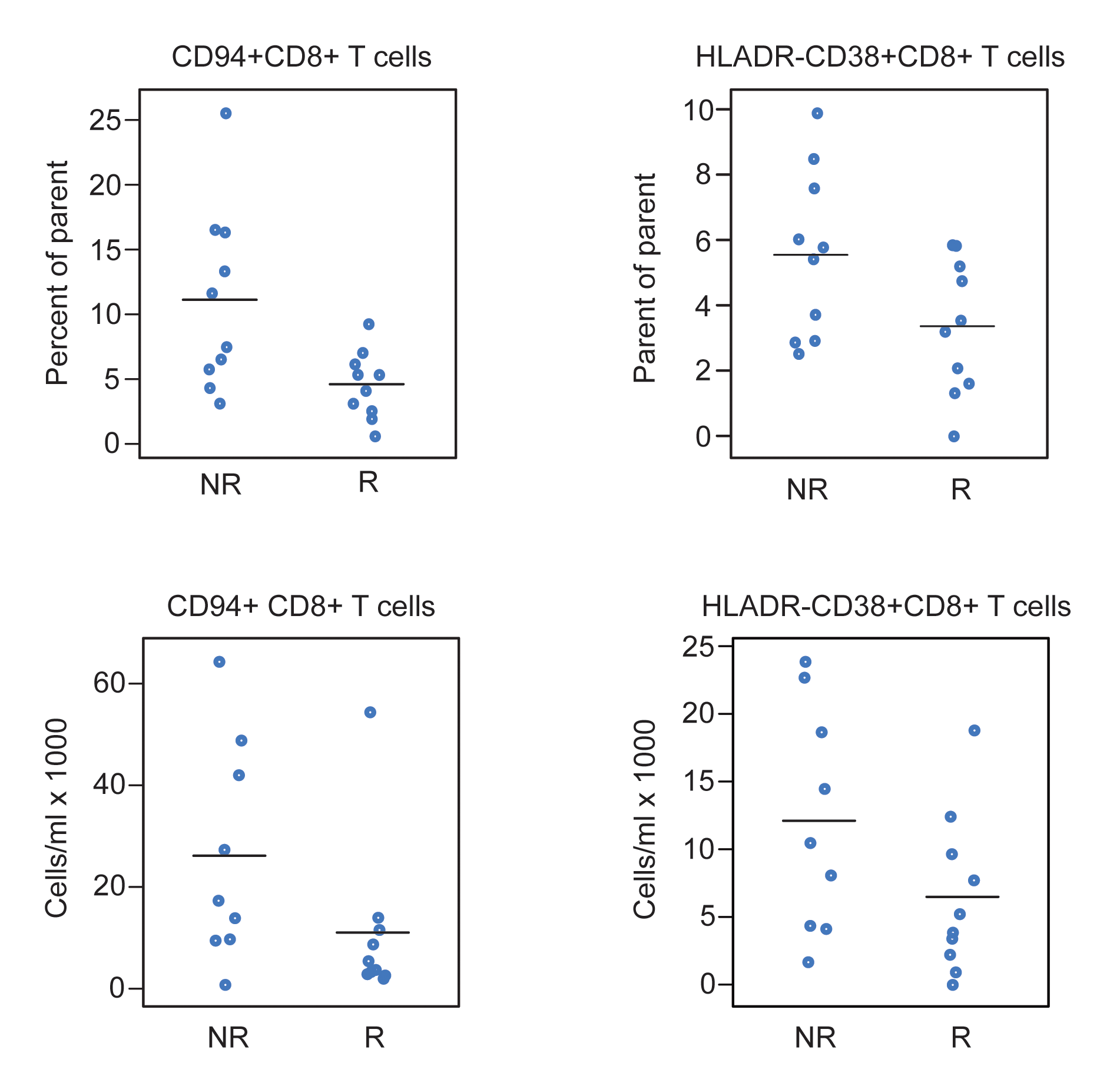

Supplement: S1 Fig — Non-responders (NR) and responders (R) show significantly different levels of CD94+CD8+ T cells and HLA-DR-CD38+CD8+ T cells at baseline (top row, n = 20). These frequency differences were concordant with differences in absolute cell counts (bottom row, n = 19), although there was no significant difference in the counts. (TIF) [file pone.0153355.s001.tif]

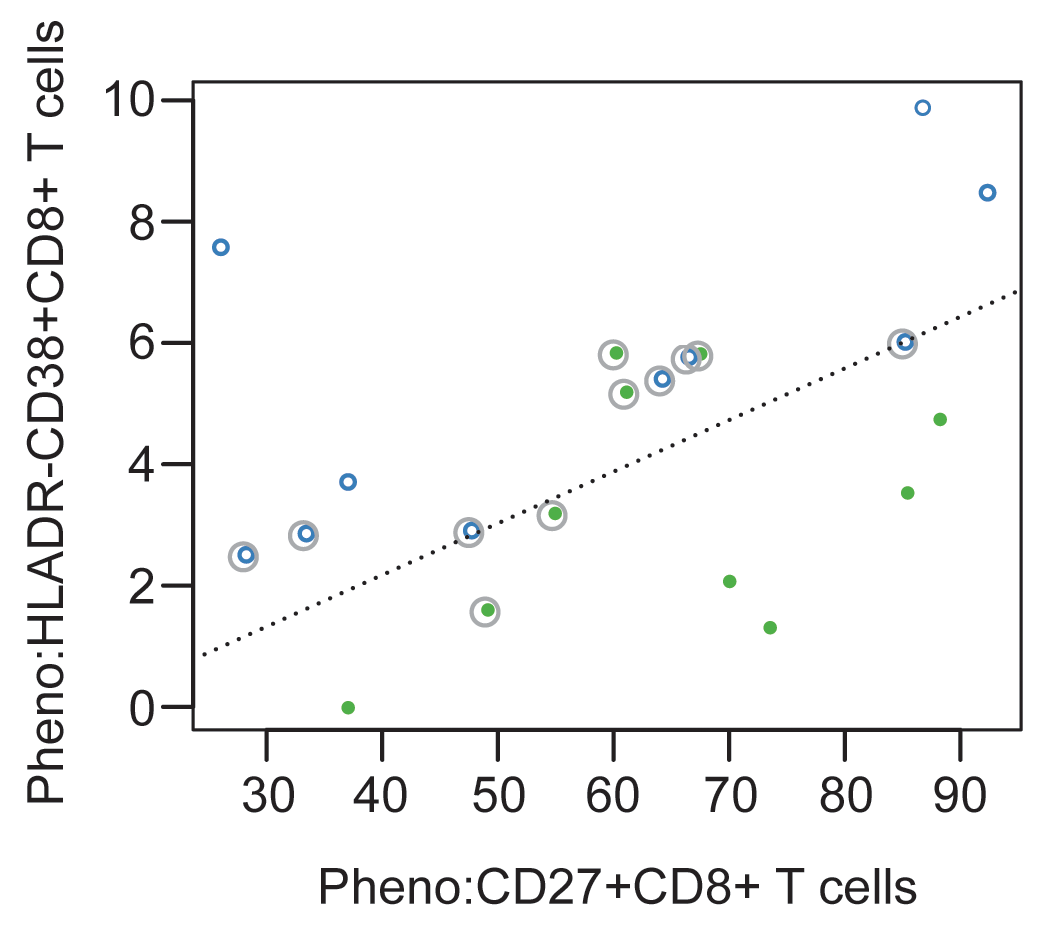

Supplement: S2 Fig — The dotted line represents the support vector, or the line that best separates responders and non-responders. The most discriminating analyte pairs, including this representative (HLA-DR-CD38+CD8+ T cells and CD27+CD8+ T cells) misclassify three samples (green points above the dotted line) in this example. Pheno = CyTOF phenotyping. (TIF) [file pone.0153355.s002.tif]

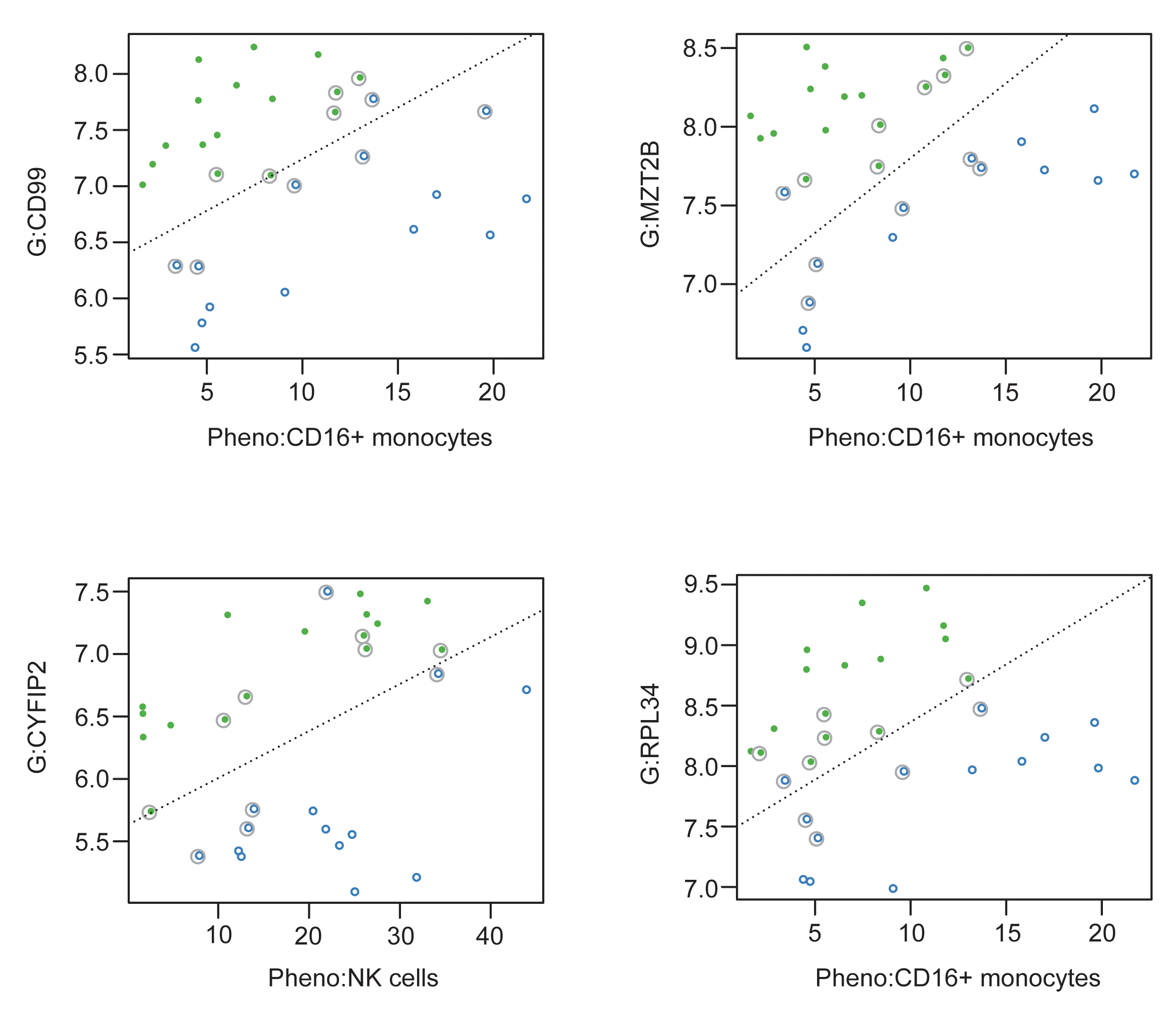

Supplement: S3 Fig — Four pairs of analytes yielding an SVM with one classification error. The dotted line represents the support vector, or the line that best separates responders and non-responders. Observations that are circled are the “support vectors,” the observations that drive the placement of the line of separation. G = gene expression; Pheno = CyTOF phenotyping. (TIF) [file pone.0153355.s003.tif]
