# Supplementary material for: Immune Profiles to Predict Response to Desensitization Therapy in Highly HLA-Sensitized Kidney Transplant Candidates
Source: PLoS One. 2016 Apr 14;11(4):e0153355. doi: 10.1371/journal.pone.0153355 (PMC4831845; doi:10.1371/journal.pone.0153355)
Supplement: S1 Table — (DOCX) [file pone.0153355.s004.docx]

**Table S1. CyTOF Antibody Immunophenotyping Panel**

| **Metal label** | **Specificity** | **Clone** |
| --- | --- | --- |
| 113In | CD57 | HCD57, BioLegend |
| 115In | live/dead |  |
| 142Nd | CD19 | SJ25C1, Southern BioTech |
| 143Nd | CD4 | SK3, BioLegend |
| 144Nd | CD8 | SK1, BioLegend |
| 146Nd | IgD | IA6-2, BioLegend |
| 147Sm | CD85j | 292319, R&D Systems |
| 148Nd | CD11c | Bu15, BioLegend |
| 149Sm | CD16 | 3G8, BioLegend |
| 150Nd | CD3 | UCHT1, BD |
| 151Eu | CD38 | HB-7, BD |
| 152Sm | CD27 | L128, BD |
| 153Eu | CD11b | ICRF44, BioLegend |
| 154Sm | CD14 | M5E2, BioLegend 11A9,  BD or G034E3 |
| 155Gd | CCR6 | BioLegend |
| 156Gd | CD94 | HP-3D9, BD |
| 157Gd | CD86 | IT2.2, BioLegend |
| 158Gd | CXCR5 | RF8B2, BD |
| 159Tb | CXCR3 | G025H7, Biolegend |
| 160Gd | CCR7 | 150503, R&D Systems |
| 162Dy | CD45RA | HI100, BioLegend |
| 164Dy | CD20 | 2H7, BioLegend |
| 165Ho | CD127 | A019D5, BioLegend |
| 166Er | CD33 | P67.8, BD |
| 167Er | CD28 | L293, BD |
| 168Er | CD24 | ML5, BioLegend |
| 169Tm | ICOS | DX29, BD |
| 170Er | CD161 | DX12, BD |
| 171Yb | TCRgd | B1, BioLegend |
| 172Yb | PD-1 | EH12.1, BD |
| 173Yb | CD123 | 9F5, BD |
| 174Yb | CD56 | NCAM16.2, BD |
| 175Lu | HLA-DR | G46-6, BD |
| 176Yb | CD25 | M-A251, BD |
