# Supplementary material for: Immune Profiles to Predict Response to Desensitization Therapy in Highly HLA-Sensitized Kidney Transplant Candidates
Source: PLoS One. 2016 Apr 14;11(4):e0153355. doi: 10.1371/journal.pone.0153355 (PMC4831845; doi:10.1371/journal.pone.0153355)
Supplement: S2 Table — (DOCX) [file pone.0153355.s005.docx]

**Table S2. CyTOF Cell Subsets and Gating Pathway**

| **Cell Subset** | **Gating Pathway** |
| --- | --- |
| B cells | CD14-CD33-/CD3-/CD19+CD20+ |
| CD16+ monocytes | CD14+CD33+/CD16+ |
| CD161+ NK cells | CD14-CD33-/CD3-/CD16+CD56+/CD161+ |
| CD161+CD4+ T cells | CD14-CD33-/CD3+/CD4+/CD161+ |
| CD161+CD45RA+ Tregs | CD14-CD33-/CD3+/CD4+/CD25hiCD127low/CD161+CD45RA+ |
| CD161+CD45RA- Tregs | CD14-CD33-/CD3+/CD4+/CD25hiCD127low/CD161+CD45RA- |
| CD161+CD8+ T cells | CD14-CD33-/CD3+/CD8+/CD161+ |
| CD161-CD45RA+ Tregs | CD14-CD33-/CD3+/CD4+/CD25hiCD127low/CD161-CD45RA+ |
| CD161-CD45RA- Tregs | CD14-CD33-/CD3+/CD4+/CD25hiCD127low/CD161-CD45RA- |
| CD27+CD8+ T cells | CD14-CD33-/CD3+/CD8+/CD27+ |
| CD28-CD8+ T cells | CD14-CD33-/CD3+/CD8+/CD28- |
| CD4+ T cells | CD14-CD33-/CD3+/CD4+ |
| CD4+CD27+ T cells | CD14-CD33-/CD3+/CD4+/CD27+ |
| CD4+CD28- T cells | CD14-CD33-/CD3+/CD4+/CD28- |
| CD8+ T cells | CD14-CD33-/CD3+/CD8+ |
| CD85j+CD4+ T cells | CD14-CD33-/CD3+/CD4+/CD85j+ |
| CD85j+CD8+ T cells | CD14-CD33-/CD3+/CD8+/CD85j+ |
| CD94+ NK cells | CD14-CD33-/CD3-/CD16+CD56+/CD94+ |
| CD94+CD4+ T cells | CD14-CD33-/CD3+/CD4+/CD94+ |
| CD94+CD8+ T cells | CD14-CD33-/CD3+/CD8+/CD94+ |
| HLADR+ NK cells | CD14-CD33-/CD3-/CD16+CD56+/HLADR+ |
| HLADR+CD38+CD4+ T cells | CD14-CD33-/CD3+/CD4+/HLADR+CD38+ |
| HLADR+CD38+CD8+ T cells | CD14-CD33-/CD3+/CD8+/HLADR+CD38+ |
| HLADR-CD38+CD4+ T cells | CD14-CD33-/CD3+/CD4+/HLADR-CD38+ |
| HLADR-CD38+CD8+ T cells | CD14-CD33-/CD3+/CD8+/HLADR-CD38+ |
| IgD+CD27+ B cells | CD14-CD33-/CD3-/CD19+CD20+/IgD+CD27+ |
| IgD+CD27- B cells | CD14-CD33-/CD3-/CD19+CD20+/IgD+CD27- |
| IgD-CD27+ B cells | CD14-CD33-/CD3-/CD19+CD20+/IgD-CD27+ |
| IgD-CD27- B cells | CD14-CD33-/CD3-/CD19+CD20+/IgD-CD27- |
| NK cells | CD14-CD33-/CD3-/CD16+CD56+ |
| NKT cells | CD14-CD33-/CD3+/CD56+ |
| T cells | CD14-CD33-/CD3+ |
| Tregs | CD14-CD33-/CD3+/CD4+/CD25hiCD127low |
| central memory CD4+ T cells | CD14-CD33-/CD3+/CD4+/CCR7+CD45RA- |
| central memory CD8+ T cells | CD14-CD33-/CD3+/CD8+/CCR7-CD45RA- |
| effector CD4+ T cells | CD14-CD33-/CD3+/CD4+/CCR7-CD45RA+ |
| effector CD8+ T cells | CD14-CD33-/CD3+/CD8+/CCR7-CD45RA+ |
| effector memory CD4+ T cells | CD14-CD33-/CD3+/CD4+/CCR7-CD45RA- |
| effector memory CD8+ T cells | CD14-CD33-/CD3+/CD8+/CCR7-CD45RA- |
| gamma-delta T cells | CD14-CD33-/TCRgd+ |
| lymphocytes | CD14-CD33- |
| monocytes | CD14+CD33+ |
| naive B cells | CD14-CD33-/CD3-/CD19+CD20+/CD24-CD38+ |
| naive CD4+ T cells | CD14-CD33-/CD3+/CD4+/CCR7+CD45RA+ |
| naive CD8+ T cells | CD14-CD33-/CD3+/CD8+/CCR7+CD45RA+ |
| plasmablasts | CD14-CD33-/CD3-/CD20-/CD27+CD38+ |
| transitional B cells | CD14-CD33-/CD3-/CD19+CD20+/CD24+CD38+ |
| viable/singlets | viable/singlets |
